# *de novo* metastases in patients with primary colorectal cancer: a Surveillance, Epidemiology, and End Results analysis

**DOI:** 10.1007/s10552-025-02002-6

**Published:** 2025-04-19

**Authors:** Nicole C. Loroña, Kamya Sankar, Mariana C. Stern, Stephanie L. Schmit, Jane C. Figueiredo

**Affiliations:** 1 Department of Medicine, Samuel Oschin Comprehensive Cancer Institute, Cedars Sinai Medical Center, 700 N. San Vicente Blvd. Suite G599, West Hollywood, Los Angeles, CA 90069 USA; 2 Department of Preventive Medicine, Keck School of Medicine, University of Southern California/Norris Comprehensive Cancer Center, Los Angeles, CA USA; 3 Genomic Medicine Institute, Cleveland Clinic, Cleveland, OH USA; 4 Department of Molecular Medicine, Cleveland Clinic Lerner College of Medicine of Case Western Reserve University, Cleveland, OH USA; 5 Department of Computational Biomedicine, Samuel Oschin Comprehensive Cancer Institute, Cedars Sinai Medical Center, Los Angeles, CA USA

**Keywords:** Disparities, Race, Ethnicity, Early-onset, Colorectal cancer, Metastatic

## Abstract

**Purpose:**

Nearly one-quarter of colorectal cancer (CRC) cases present with *de novo* metastatic disease (stage IV) at diagnosis. Some metastatic sites confer poorer prognosis, and emerging data suggests that individuals from certain racial and ethnic populations may be at higher risk for *de novo* metastases.

**Methods:**

We identified 181,083 CRC cases aged 20–84 between 2010 and 2020 in the Surveillance, Epidemiology, and End Results database. Two outcomes were analyzed: metastatic site (liver, lung, bone, brain) and metastatic pattern (liver only, lung only, liver and lung, other). We used logistic and multinomial logistic regression to calculate odds ratios (OR) and 95% confidence intervals (95% CI) to examine the associations between race and ethnicity and metastatic site and metastatic pattern, respectively, among early-onset (age < 50), screen-eligible (age 50–74), and elderly populations (age 75–84).

**Results:**

43,054 *de novo* metastatic CRC cases were identified over the 10-year period. Liver was the most common metastatic site (80%). Non-Hispanic Black patients had higher odds of synchronous lung and liver metastases compared to non-Hispanic White (NHW) patients (early-onset patients: OR: 1.29, 95% CI 1.03–1.61; screen-eligible patients: OR:1.42, 95% CI 1.29–1.55; elderly patients: OR:1.66, 95% CI 1.34–2.05). Early-onset American Indian/Alaska Native patients were over twice as likely to present with lung metastases (OR: 2.10, 95% CI 1.11–3.98) compared to NHW patients.

**Conclusions:**

Presentation and patterns of *de novo* metastatic CRC differed across populations and age groups. Characterizing *de novo* metastatic CRC provides a unique opportunity to study cancer spread in treatment-naïve individuals and to identify patients at greater risk of metastases associated with poorer prognosis.

## Introduction

Colorectal cancer (CRC) is currently the first and second cause of cancer-specific mortality in men and women under age 50, respectively [1]. Cancer-specific mortality is largely driven by metastatic spread [2]. About 15–25% of patients with stage I–III CRC will develop distant metastases in the five years following initial diagnosis, with the most common distant metastatic site being the liver [3–5]. Overall, 21% percent of CRCs are metastatic at diagnosis (*de novo* metastatic CRC) [6]. Clinical treatment of metastatic CRC is dependent upon the site of metastasis [5]. For example, resection of liver metastases may be curative in some instances of resectable metastatic CRC, while survival may be prolonged in cases of non-resectable metastatic CRC using a combination of chemotherapy, targeted treatments, and immunotherapy [5, 7].

The liver is the most common site of distant metastasis in CRC due to the mesenteric drainage from the large intestine into the hepatic portal venous system. However, factors influencing spread to the liver and other sites are not fully understood [8]. Metastatic organotropism (non-random metastatic spread to specific sites) in CRC may be driven by tumor cell intrinsic factors, the tumor microenvironment, and their interaction [9, 10]. *de novo* metastatic CRC provides a unique opportunity to study how CRC spreads in treatment-naïve individuals and to increase understanding of population groups at greater risk of metastases associated with poorer prognosis.

Significant racial and ethnic disparities in CRC mortality have persisted over decades. Black, American Indian, and Alaska Native individuals experience higher mortality rates than non-Hispanic White individuals [1]. Disparities in CRC mortality are driven by a complexity of multilevel factors, such as access to care, stage at diagnosis, tumor biology, and treatment efficacy [11]. With prognosis of metastatic CRC dependent on the site of metastasis, differing propensity of spread to distant sites may also be a factor influencing disparities in CRC mortality. Previous research using data from the Surveillance, Epidemiology, and End Results (SEER) program has found that site and patterns of de novo CRC metastases differ by race and ethnicity in non-Hispanic Black, non-Hispanic White, and Hispanic adults aged 40 and above [12]. Specifically, non-Hispanic Black patients were more likely to have metastases at diagnosis, with a 30% greater odds of *de novo* liver metastases and a 24% greater odds of *de novo* lung metastases after adjustment for age, marital status, and sex [12]. Heterogeneity in molecular features and mutational status of CRC by race and ethnicity have been observed overall and in early-onset CRC [13, 14], and this heterogeneity may partly contribute to differences in sites and patterns of *de novo* metastases. Characterizing differences in *de novo* metastatic CRC by race, ethnicity, and age can inform surveillance strategies in patients with stage I–III CRC in an effort to improve CRC outcomes across populations.

In this analysis, we comprehensively examine patterns of *de novo* metastases across additional racial and ethnic groups (including Non-Hispanic White, Hispanic, non-Hispanic American Indian/Alaska Native, non-Hispanic Asian or Pacific Islander, and non-Hispanic Black (NHB))) and among adults aged 20–84 using data from the population-based registries within SEER. Furthermore, we examined associations separately for early-onset, screen-eligible and elderly populations.

## Methods

### Study population

We identified 244,655 CRC patients listed in the Surveillance, Epidemiology, and End Results (SEER) 17 registries database[15] diagnosed with a first primary invasive CRC (excluding appendiceal tumors) from 2010 to 2020, aged 20–84 at diagnosis, excluding those diagnosed only by autopsy or death certificate. Beginning in 2010, the SEER program has collected information on metastases to the liver, lung, bone, and brain present at primary cancer diagnosis. The present study population includes individuals with both non-metastatic and metastatic disease at diagnosis.

### Outcomes

Patients presenting with stage IV CRC at diagnosis were classified as having *de novo* metastatic CRC. Outcomes included non-mutually exclusive site of *de novo* metastasis (defined as metastases of the liver, lung, bone or brain present at the time of diagnosis, i.e., stage IV), coded as individual binary variables (i.e. none, present for each metastatic location) and mutually exclusive pattern of *de novo* metastasis (liver only, lung only, synchronous liver and lung, other). For each outcome, no metastasis at diagnosis (stage I–III CRC) was the reference category. SEER does not collect data on recurrence or metastasis occurring after the initial cancer diagnosis.

### Covariates

Analyses were stratified by three age groups: early-onset (age at diagnosis 20–49), screen-eligible (50–74), and elderly (75–84). Age at diagnosis was included as a continuous variable in all models. We used the SEER-provided variable for race and ethnicity with six mutually exclusive groups: Non-Hispanic White (NHW), Hispanic (all races), non-Hispanic American Indian/Alaska Native (AI/AN), non-Hispanic Asian or Pacific Islander (API), non-Hispanic Black (NHB), and non-Hispanic unknown race (NHUR). Due to small sample size, NHUR cases were excluded from analyses. Anatomic site of primary CRC was classified as right colon (cecum through transverse colon), left colon (splenic flexure through sigmoid colon), rectosigmoid junction, or rectum. Grade (1, 2, 3, 4), tumor (T) stage (T0–T2, T3, T4, TX), and node (N) stage (N0, N1, N2, NX) were included in models as categorical (dummy) variables. County-level median household income (< $35,000, $35,000–$44,999, $45,000–$54,999, $55,000–$64,999, $65,000–$74,999, ≥ $75,000) was provided by SEER based on American Community Survey estimates inflation-adjusted to 2021. Urban/rural county residence was derived by SEER using Rural–Urban Continuum Codes.

### Statistical analysis

Patients with stage 0 disease (*n* = 6,745) or missing data on SEER-provided summary stage at diagnosis (*n* = 18,997), race or ethnicity (*n* = 1,261), age (*n* = 8,437), anatomic site (*n* = 6,798), T stage or N stage (*n* = 185), county-level median household income (*n* = 20,606), or urban/rural county residence (*n* = 543) were excluded in a stepwise fashion resulting in a final analytic sample of 181,083 patients. Logistic regression models were used to calculate odds ratios (OR) and 95% confidence intervals (95% CI) for the associations between racial and ethnic group and each site of metastasis stratified by age group (20–49, 50–74, 75 +). Multinomial logistic regression was used to assess the association between racial and ethnic group and pattern of metastasis stratified by age group. NHW was the reference racial and ethnic group. Models were adjusted for continuous age, sex, site and side (right colon, left colon, rectosigmoid, rectum), T stage, N stage, county-level median household income (< $35,000, $35,000–$44,999, $45,000–$54,999, $55,000–$64,999, $65,000–$74,999, ≥ $75,000), and urban/rural county residence. Interaction between age group and racial and ethnic group (interaction terms with age group and race and ethnicity) in each model was assessed using a likelihood ratio test. All tests were two-sided, and *p*-values less than 0.05 were considered statistically significant. All analyses were performed using R version 4.3.1 [16].

## Results

### Descriptive statistics

Between 2010 and 2020, 138,029 stage I–III CRC cases and 43,054 *de novo* metastatic CRC cases were diagnosed, after exclusions for missing data. Patients with metastatic disease at diagnosis had a lower mean age, and were generally more likely to be male and NHB than patients with non-metastatic disease (Table 1). Patients with liver only or synchronous lung and liver metastases at diagnosis were more likely to be male, NHB, and have primary tumors in the left colon or rectosigmoid junction (Table 2). The liver was the most common site of metastasis (*n* = 32,175; 80% of those with metastases) followed by the lung (*n* = 11,225; 30%). Of metastatic cases, there were 7% (*n* = 2,662) with metastases to the lung only, 55% (*n* = 22,028) to the liver only, 17% (*n* = 6,834) with synchronous lung and liver metastases, and 22% (*n* = 8,643) with other site/pattern of metastasis.

**Table 1:** Distribution of demographic and clinical characteristics by site of distant metastasis; Surveillance, Epidemiology, and End Results 17 Registries 2010–2020

	Not Stage IV (*n* = 138,029)	Liver (*n* = 32,175)	Lung (*n* = 11,225)	Bone (*n* = 2,641)	Brain (*n* = 581)
Age at diagnosis					
Mean (SD)	63.4 (11.9)	62.1 (11.9)	62.8 (11.6)	61.7 (11.7)	62.6 (11.2)
20–49	14,780 (10.7%)	4,457 (13.9%)	1,367 (12.2%)	365 (13.8%)	67 (11.5%)
50–74	95,080 (68.9%)	22,400 (69.6%)	7,928 (70.6%)	1,889 (71.5%)	421 (72.5%)
75–84	28,169 (20.4%)	5,318 (16.5%)	1,930 (17.2%)	387 (14.7%)	93 (16.0%)
Sex					
Female	63,976 (46.3%)	13,408 (41.7%)	4,944 (44.0%)	1,028 (38.9%)	293 (50.4%)
Male	74,053 (53.7%)	18,767 (58.3%)	6,281 (56.0%)	1,613 (61.1%)	288 (49.6%)
Race and ethnicity					
Non-Hispanic White	88,347 (64.0%)	19,998 (62.2%)	6,920 (61.6%)	1,644 (62.2%)	384 (66.1%)
Hispanic (all races)	18,930 (13.7%)	4,267 (13.3%)	1,479 (13.2%)	352 (13.3%)	80 (13.8%)
Non-Hispanic American Indian/Alaska Native	922 (0.7%)	208 (0.6%)	96 (0.9%)	19 (0.7%)	6 (1.0%)
Non-Hispanic Asian or Pacific Islander	14,332 (10.4%)	2,932 (9.1%)	1,093 (9.7%)	254 (9.6%)	38 (6.5%)
Non-Hispanic Black	15,498 (11.2%)	4,770 (14.8%)	1,637 (14.6%)	372 (14.1%)	73 (12.6%)
Anatomic site of primary cancer					
Right colon	53,317 (38.6%)	11,902 (37.0%)	3,438 (30.6%)	786 (29.8%)	223 (38.4%)
Left colon	38,129 (27.6%)	9,849 (30.6%)	2,989 (26.6%)	670 (25.4%)	144 (24.8%)
Rectosigmoid junction	10,947 (7.9%)	3,289 (10.2%)	1,240 (11.0%)	298 (11.3%)	63 (10.8%)
Rectum	35,636 (25.8%)	7,135 (22.2%)	3,558 (31.7%)	887 (33.6%)	151 (26.0%)
Grade					
1	14,013 (10.2%)	1,348 (4.2%)	512 (4.6%)	97 (3.7%)	13 (2.2%)
2	83,317 (60.4%)	15,467 (48.1%)	5,283 (47.1%)	922 (34.9%)	225 (38.7%)
3	14,143 (10.2%)	4,735 (14.7%)	1,393 (12.4%)	560 (21.2%)	110 (18.9%)
4	2,533 (1.8%)	720 (2.2%)	180 (1.6%)	64 (2.4%)	15 (2.6%)
Missing	24,023 (17.4%)	9,905 (30.8%)	3,857 (34.4%)	998 (37.8%)	218 (37.5%)
T stage					
T0–T2	51,357 (37.2%)	3,500 (10.9%)	1,301 (11.6%)	326 (12.3%)	67 (11.5%)
T3	67,882 (49.2%)	10,003 (31.1%)	2,896 (25.8%)	524 (19.8%)	135 (23.2%)
T4	18,096 (13.1%)	7,466 (23.2%)	2,450 (21.8%)	585 (22.2%)	105 (18.1%)
TX	694 (0.5%)	11,206 (34.8%)	4,578 (40.8%)	1206 (45.7%)	274 (47.2%)
N stage					
N0	86,200 (62.5%)	9,620 (29.9%)	3,464 (30.9%)	799 (30.3%)	169 (29.1%)
N1	36,376 (26.4%)	9,111 (28.3%)	3,021 (26.9%)	620 (23.5%)	131 (22.5%)
N2	15,301 (11.1%)	6,636 (20.6%)	1,718 (15.3%)	413 (15.6%)	102 (17.6%)
NX	152 (0.1%)	6,808 (21.2%)	3,022 (26.9%)	809 (30.6%)	179 (30.8%)
Urban/rural county residence					
Urban	119,057 (86.3%)	27,682 (86.0%)	9,654 (86.0%)	2,296 (86.9%)	478 (82.3%)
Rural	18,972 (13.7%)	4,493 (14.0%)	1,571 (14.0%)	345 (13.1%)	103 (17.7%)
County-level median household income^a^					
< $35,000	2,105 (1.5%)	517 (1.6%)	176 (1.6%)	35 (1.3%)	17 (2.9%)
$35,000–$44,999	9,540 (6.9%)	2,313 (7.2%)	793 (7.1%)	180 (6.8%)	39 (6.7%)
$45,000–$54,999	17,011 (12.3%)	4,053 (12.6%)	1,421 (12.7%)	322 (12.2%)	84 (14.5%)
$55,000–$64,999	12,985 (9.4%)	3,078 (9.6%)	1,034 (9.2%)	259 (9.8%)	56 (9.6%)
$65,000–$74,999	36,905 (26.7%)	8,535 (26.5%)	2,993 (26.7%)	726 (27.5%)	153 (26.3%)
$75,000 +	59,483 (43.1%)	13,679 (42.5%)	4,808 (42.8%)	1,119 (42.4%)	232 (39.9%)

*SD* standard deviation

^a^County-level Median Household Income in 2021 inflation-adjusted dollars

**Table 2:** Distribution of demographic and clinical characteristics by pattern of distant metastasis; Surveillance, Epidemiology, and End Results 17 Registries 2010–2020

	Not stage IV (*n* = 138,029)	Lung only (*n* = 2,662)	Liver only (*n* = 22,028)	Lung + liver (*n* = 6,834)	Stage IV, other (*n* = 8,643)
Age at diagnosis					
Mean (SD)	63.4 (11.9)	63.8 (11.9)	61.9 (12.1)	62.4 (11.6)	61.9 (12.7)
20–49	14,780 (10.7%)	311 (11.7%)	3,192 (14.5%)	855 (12.5%)	1,333 (15.4%)
50–74	95,080 (68.9%)	1,801 (67.7%)	15,160 (68.8%)	4,877 (71.4%)	5,764 (66.7%)
75–84	28,169 (20.4%)	550 (20.7%)	3,676 (16.7%)	1,102 (16.1%)	1,546 (17.9%)
Sex					
Female	63,976 (46.3%)	1,299 (48.8%)	9,182 (41.7%)	2,909 (42.6%)	4,148 (48.0%)
Male	74,053 (53.7%)	1,363 (51.2%)	12,846 (58.3%)	3,925 (57.4%)	4,495 (52.0%)
Race and ethnicity					
Non-Hispanic White	88,347 (64.0%)	1,644 (61.8%)	13,758 (62.5%)	4,210 (61.6%)	5,416 (62.7%)
Hispanic (all races)	18,930 (13.7%)	371 (13.9%)	2,900 (13.2%)	863 (12.6%)	1,232 (14.3%)
Non-Hispanic American Indian/Alaska Native	922 (0.7%)	24 (0.9%)	133 (0.6%)	55 (0.8%)	64 (0.7%)
Non-Hispanic Asian or Pacific Islander	14,332 (10.4%)	288 (10.8%)	1,972 (9.0%)	639 (9.4%)	815 (9.4%)
Non-Hispanic Black	15,498 (11.2%)	335 (12.6%)	3,265 (14.8%)	1,067 (15.6%)	1,116 (12.9%)
Anatomic site					
Right colon	53,317 (38.6%)	728 (27.3%)	8,563 (38.9%)	2,197 (32.1%)	3,764 (43.5%)
Left colon	38,129 (27.6%)	632 (23.7%)	7,020 (31.9%)	1,927 (28.2%)	2,323 (26.9%)
Rectosigmoid junction	10,947 (7.9%)	265 (10.0%)	2,148 (9.8%)	765 (11.2%)	780 (9.0%)
Rectum	35,636 (25.8%)	1,037 (39.0%)	4,297 (19.5%)	1,945 (28.5%)	1,776 (20.5%)
Grade					
1	14,013 (10.2%)	139 (5.2%)	909 (4.1%)	306 (4.5%)	348 (4.0%)
2	83,317 (60.4%)	1,361 (51.1%)	10,967 (49.8%)	3,211 (47.0%)	3,158 (36.5%)
3	14,143 (10.2%)	341 (12.8%)	3,393 (15.4%)	788 (11.5%)	2,173 (25.1%)
4	2,533 (1.8%)	59 (2.2%)	549 (2.5%)	93 (1.4%)	378 (4.4%)
Missing	24,023 (17.4%)	762 (28.6%)	6,210 (28.2%)	2,436 (35.6%)	2,586 (29.9%)
T stage					
T0–T2	51,357 (37.2%)	289 (10.9%)	2,294 (10.4%)	830 (12.1%)	650 (7.5%)
T3	67,882 (49.2%)	967 (36.3%)	7,780 (35.3%)	1,603 (23.5%)	2,234 (25.8%)
T4	18,096 (13.1%)	704 (26.4%)	5,403 (24.5%)	1,407 (20.6%)	3,668 (42.4%)
TX	694 (0.5%)	702 (26.4%)	6,551 (29.7%)	2,994 (43.8%)	2,091 (24.2%)
N stage					
N0	86,200 (62.5%)	859 (32.3%)	6,601 (30.0%)	2,131 (31.2%)	2,232 (25.8%)
N1	36,376 (26.4%)	809 (30.4%)	6,506 (29.5%)	1,818 (26.6%)	2,375 (27.5%)
N2	15,301 (11.1%)	470 (17.7%)	5,124 (23.3%)	1,036 (15.2%)	2,583 (29.9%)
NX	152 (0.1%)	524 (19.7%)	3,797 (17.2%)	1,849 (27.1%)	1,453 (16.8%)
Urban/rural county residence					
Urban	119,057 (86.3%)	2,280 (85.6%)	18,930 (85.9%)	5,874 (86.0%)	7,435 (86.0%)
Rural	18,972 (13.7%)	382 (14.4%)	3,098 (14.1%)	960 (14.0%)	1,208 (14.0%)
County-level median household income^a^					
< $35,000	2,105 (1.5%)	46 (1.7%)	369 (1.7%)	103 (1.5%)	140 (1.6%)
$35,000–$44,999	9,540 (6.9%)	213 (8.0%)	1,640 (7.4%)	482 (7.1%)	644 (7.5%)
$45,000–$54,999	17,011 (12.3%)	329 (12.4%)	2,806 (12.7%)	869 (12.7%)	1,103 (12.8%)
$55,000–$64,999	12,985 (9.4%)	255 (9.6%)	2,135 (9.7%)	637 (9.3%)	810 (9.4%)
$65,000-$74,999	36,905 (26.7%)	689 (25.9%)	5,757 (26.1%)	1,774 (26.0%)	2,330 (27.0%)
$75,000 +	59,483 (43.1%)	1,130 (42.4%)	9,321 (42.3%)	2,969 (43.4%)	3,616 (41.8%)

*SD* standard deviation

^a^County-level Median Household Income in 2021 inflation-adjusted dollars

### *de novo metastatic CRC among individuals under age 50*

Within the 20–49 age group, Hispanic patients were less likely to have liver metastases than NHW patients (OR: 0.86, 95% CI 0.77–0.96). AI/AN patients were over twice as likely to present with lung metastases (OR: 2.10, 95% CI 1.11–3.98) than NHW patients (Table 3). API individuals were more likely to have lung (OR: 1.26, 95% CI 1.00–1.59) *de novo* metastases than NHW patients. Hispanic and API patients had lower odds of a liver-only metastatic pattern than NHW patients (Table 4). NHB individuals had greater odds of synchronous liver and lung (OR: 1.29, 95% CI 1.03–1.61) metastases than NHW patients.

**Table 3:** Risks of *de novo* liver, lung, bone, and brain metastases, relative to risk of non-metastatic colorectal cancer, by race and ethnicity within three age at diagnosis groups

	Not stage IV (*n* = 138,029)	Liver (*n* = 32,175)		Lung (*n* = 11,225)		Bone (*n* = 2,641)		Brain (*n* = 581)	
	*n* (%)	*n* (%)	OR (95% CI)	*n* (%)	OR (95% CI)	*n* (%)	OR (95% CI)	*n* (%)	OR (95% CI)
Age 20–49 at diagnosis									
Non-Hispanic White	8,122 (76.7)	2,471 (23.3)	Reference	702 (8.0)	Reference	177 (2.1)	Reference	36 (0.4)	Reference
Hispanic (all races)	2,957 (77.6)	853 (22.4)	0.86 (0.77, 0.96)*	270 (8.4)	1.00 (0.82, 1.22)	78 (2.6)	1.29 (0.91, 1.83)	15 (0.5)	1.28 (0.61, 2.69)
Non-Hispanic AI/AN^2^	120 (76.9)	36 (23.1)	1.18 (0.75, 1.85)	15 (11.1)	2.10 (1.11, 3.98)*	a	a	a	a
Non-Hispanic API^3^	1,664 (78.3)	462 (21.7)	0.91 (0.79, 1.05)	168 (9.2)	1.26 (1.00, 1.59)*	52 (3.2)	1.51 (0.99, 2.30)	a	a
Non-Hispanic black	1,917 (75.1)	635 (24.9)	1.03 (0.90, 1.17)	212 (10.0)	1.22 (0.98, 1.52)	54 (2.7)	1.36 (0.90, 2.05)	a	a
Age 50–74 at diagnosis									
Non-Hispanic White	59,916 (81.2)	13,896 (18.8)	Reference	4,906 (7.6)	Reference	1191 (1.9)	Reference	282 (0.5)	Reference
Hispanic (all races)	13,159 (82.0)	2,884 (18.0)	0.89 (0.84, 0.94)*	998 (7.0)	0.90 (0.82, 0.99)*	234 (1.7)	0.88 (0.73, 1.05)	54 (0.4)	0.93 (0.66, 1.31)
Non-Hispanic AI/AN^2^	661 (81.9)	146 (18.1)	0.85 (0.67, 1.08)	67 (9.2)	1.07 (0.76, 1.51)	13 (1.9)	0.77 (0.38, 1.56)	a	a
Non-Hispanic API^3^	10,022 (83.4)	1,995 (16.6)	0.86 (0.80, 0.92)*	755 (7.0)	0.89 (0.80, 0.99)*	169 (1.7)	0.78 (0.63, 0.96)*	22 (0.2)	0.40 (0.24, 0.69)*
Non-Hispanic Black	11,322 (76.5)	3,479 (23.5)	1.29 (1.22, 1.36)*	1,202 (9.6)	1.27 (1.16, 1.39)*	282 (2.4)	1.20 (1.01, 1.43)*	58 (0.5)	0.97 (0.69, 1.37)
Age 75–84 at diagnosis									
Non-Hispanic White	20,309 (84.8)	3,631 (15.2)	Reference	1,312 (6.1)	Reference	276 (1.3)	Reference	66 (0.3)	Reference
Hispanic (all races)	2,814 (84.2)	530 (15.8)	0.96 (0.84, 1.10)	211 (7.0)	1.07 (0.87, 1.32)	40 (1.4)	0.99 (0.65, 1.51)	11 (0.4)	1.00 (0.44, 2.27)
Non-Hispanic AI/AN^2^	141 (84.4)	26 (15.6)	0.75 (0.41, 1.36)	14 (9.0)	1.97 (1.01, 3.85)*	a	a	a	a
Non-Hispanic API^3^	2,646 (84.8)	475 (15.2)	0.98 (0.85, 1.12)	170 (6.0)	1.08 (0.87, 1.35)	33 (1.2)	0.81 (0.50, 1.31)	a	a
Non-Hispanic Black	2,259 (77.5)	656 (22.5)	1.61 (1.42, 1.82)*	223 (9.0)	1.54 (1.26, 1.89)*	36 (1.6)	0.94 (0.58, 1.52)	a	a

*p-value < 0.05

^a^Results not shown due to cell sample size < 10

Models adjusted for: continuous age, sex, site and side (right colon, left colon, rectosigmoid, rectum), T stage, N stage, county-level median household income (< $35,000, $35,000–$44,999, $45,000–$54,999, $55,000–$64,999, $65,000–$74,999, ≥ $75,000), and urban/rural county residence. *AI/AN* American Indian/Alaska native, *API* Asian/pacific islander

**Table 4:** Risks of patterns of synchronous *de novo* metastases, relative to the risk of non-metastatic colorectal cancer, by race and ethnicity within three age at diagnosis groups

	Not stage IV (*n* = 138,029)	Lung only (*n* = 2,662)		Liver only (*n* = 22,028)		Lung + liver (*n* = 6,834)		Stage IV, other (*n* = 8,643)	
	*n* (%)	*n* (%)	OR (95% CI)	*n* (%)	OR (95% CI)	*n* (%)	OR (95% CI)	*n* (%)	OR (95% CI)
Age 20–49 at diagnosis									
Non-Hispanic White	8,122 (72.5)	155 (1.4)	Reference	1,806 (16.1)	Reference	451 (4.0)	Reference	674 (6.0)	Reference
Hispanic (all races)	2,957 (72.1)	67 (1.6)	1.19 (0.88, 1.61)	606 (14.8)	0.86 (0.77, 0.97)*	160 (5.3)	0.89 (0.73, 1.09)	310 (7.6)	1.15 (0.98, 1.34)
Non-Hispanic AI/AN^2^	120 (70.6)	a	a	23 (13.5)	1.01 (0.62, 1.66)	a	a	15 (8.8)	1.76 (0.97, 3.18)
Non-Hispanic API^3^	1,664 (73.1)	43 (1.9)	1.42 (0.99, 2.04)	308 (13.5)	0.84 (0.72, 0.98)*	98 (4.3)	1.04 (0.81, 1.34)	163 (7.2)	1.19 (0.98, 1.45)
Non-Hispanic Black	1,917 (70.6)	43 (1.6)	1.21 (0.85, 1.73)	449 (16.5)	0.99 (0.87, 1.14)	137 (5.0)	1.29 (1.03, 1.61)*	171 (6.3)	1.00 (0.82, 1.21)
Age 50–74 at diagnosis									
Non-Hispanic White	59,916 (77.7)	1,111 (1.4)	Reference	9,432 (12.2)	Reference	3,019 (3.9)	Reference	3,647 (4.7)	Reference
Hispanic (all races)	13,159 (78.8)	246 (1.5)	1.00 (0.85, 1.18)	1,946 (11.6)	0.88 (0.83, 0.94)*	582 (3.5)	0.78 (0.71, 0.87)*	773 (4.6)	0.92 (0.84, 1.00)*
Non-Hispanic AI/AN^2^	661 (78.1)	16 (1.9)	0.26 (0.10, 0.70)*	92 (10.9)	0.49 (0.37, 0.65)*	39 (4.6)	0.39 (0.25, 0.63)*	38 (4.5)	0.32 (0.20, 0.51)*
Non-Hispanic API^3^	10,022 (80.1)	195 (1.6)	1.10 (0.92, 1.31)	1344 (10.7)	0.85 (0.79, 0.91)*	445 (3.6)	0.83 (0.74, 0.93)*	510 (4.1)	0.88 (0.79, 0.98)*
Non-Hispanic Black	11,322 (73.1)	233 (1.5)	1.26 (1.06, 1.48)*	2,346 (15.1)	1.24 (1.17, 1.32)*	792 (5.1)	1.42 (1.29, 1.55)*	796 (5.1)	1.13 (1.03, 1.23)*
Age 75–84 at diagnosis									
Non-Hispanic White	20,309 (81.1)	378 (1.5)	Reference	2,520 (10.1)	Reference	740 (3.0)	Reference	1,095 (4.4)	Reference
Hispanic (all races)	2,814 (80.6)	58 (1.7)	1.00 (0.75, 1.34)	348 (10.0)	0.90 (0.78, 1.04)	121 (3.5)	1.03 (0.83, 1.29)	149 (4.3)	0.91 (0.75, 1.10)
Non-Hispanic AI/AN^2^	141 (77.5)	a	a	18 (9.9)	0.81 (0.45, 1.46)	a	a	11 (6.0)	1.08 (0.54, 2.16)
Non-Hispanic API^3^	2,646 (81.3)	50 (1.5)	0.99 (0.72, 1.36)	320 (9.8)	0.95 (0.82, 1.10)	96 (3.0)	1.01 (0.79, 1.29)	142 (4.4)	0.98 (0.80, 1.19)
Non-Hispanic Black	2,259 (73.5)	59 (1.9)	1.37 (1.02, 1.83)*	470 (15.3)	1.62 (1.42, 1.84)*	138 (4.5)	1.66 (1.34, 2.05)*	149 (4.8)	1.16 (0.95, 1.41)

*p-value < 0.05

^a^Results not shown due to cell sample size < 10

Models adjusted for: continuous age, sex, site and side (right colon, left colon, rectosigmoid, rectum), T stage, N stage, county-level median household income (< $35,000, $35,000–$44,999, $45,000–$54,999, $55,000–$64,999, $65,000–$74,999, ≥ $75,000), and urban/rural county residence. *AI/AN* American Indian/Alaska native, *API* Asian/pacific islander

### *de novo metastatic CRC among screen-eligible adults*

Patients of all racial and ethnic minority groups aged 50–74 at diagnosis generally had lower odds of *de novo* distant metastases than NHW patients, except for NHB patients who had higher odds of liver (OR: 1.29, 95% CI 1.22–1.36), lung (OR: 1.27, 95% CI 1.16–1.39), and bone (OR: 1.20, 95% CI 1.01–1.43) metastases (Table 3). Patients in this age group also had lower odds of specific metastatic patterns than NHW patients, aside from NHB patients who had higher odds of liver only (OR: 1.24, 95% CI 1.17–1.32), lung only (OR: 1.26, 95% CI 1.06–1.48), synchronous liver and lung (OR: 1.42, 95% CI 1.29, 1.55), and other (OR: 1.13, 95% CI 1.03–1.23) metastatic patterns (Table 4).

### *de novo metastatic CRC among elderly adults*

Among those aged 75–84 at diagnosis, AI/AN individuals had a nearly two times greater odds of lung metastases (OR: 1.97, 95% CI 1.01–3.85), and NHB individuals had a greater odds of liver (OR: 1.61, 95% CI 1.42–1.82) and lung (OR: 1.54, 95% CI 1.26–1.89) metastases than NHW individuals (Table 3). For metastatic pattern, only NHB patients had higher odds of liver only (OR: 1.62, 95% CI 1.42–1.84), lung only (OR: 1.37, 95% CI 1.02–1.83), and synchronous liver and lung (OR: 1.66, 95% CI 1.34–2.05) metastases than NHW patients (Table 4). There were statistically significant interactions between age group and race and ethnicity in logistic regression models for liver (*p* < 0.001) and lung metastases (*p* = 0.03), and in multinomial logistic regression models for metastatic pattern (*p* < 0.001).

## Discussion

In this population-based analysis using 10 years of data from the SEER program, we identified racial and ethnic differences in *de novo* metastatic CRC in both non-screen-eligible and screen-eligible age groups after multivariable adjustment. Age at diagnosis modified the association between race and ethnicity and metastatic site and pattern, with main effect associations of the largest magnitude observed for AI/AN individuals for lung metastases in the younger (age 20–49) and older (age 75–84) age groups. NHB patients had higher odds of nearly every metastatic site and pattern in every age group, regardless of being part of a screen-eligible age group.

Similar to our findings, a previous analysis of SEER data observed higher odds of liver and lung metastases among NHB individuals aged 40 and older, compared to NHW; however, aside from Hispanic patients, no other racial or ethnic groups were examined, and patients were not further stratified by age [12]. Previous population-based work identified a greater likelihood of *de novo* lung metastases and synchronous liver and lung metastases associated with rectal cancer (vs. proximal colon), when compared to likelihood of liver-only metastases, after adjustment for race (White, non-White) and age [17]. Anatomic site of CRC differs by age, race, and ethnicity, with rectal tumors being more common in early-onset and API patients, and proximal colon tumors most common among NHB patients [18, 19]; however, we adjusted for anatomic site and side of the primary tumor (right colon, left colon, rectosigmoid, rectum) so differences in distribution of tumor site alone are unlikely to account for our results. Our findings are also consistent with observed higher risk of being diagnosed with late-stage CRC in younger, NHB, and AI/AN individuals. Specifically, 27% of distant stage CRC diagnosed in 2022 were under the age of 50, 25% were diagnosed among NHB, and 22% among AI/AN individuals [20]. We found that NHB patients had higher odds of presenting with distant metastases at CRC diagnosis than NHW, and similarly, Black patients with CRC also have a higher risk of locoregional or distant recurrence than NHW patients, independent of receipt of guideline-concordant treatment, pointing to other explanations for observed disparities [21].

CRC is predisposed to metastasis to the liver, largely due to the drainage of blood from the intestines to the liver via the hepatic portal vein; however, interest remains in identifying factors that influence successful dissemination and invasion of metastatic tumor cells into distant sites [22]. Although the exact mechanism of tumor cell dissemination and invasion in distant tissue is still unclear, the interaction between tumor cells and target organ microenvironments can impact the success of metastatic invasion and proliferation [22–24]. Hyperlipidemia, for example, interacts with the immune microenvironment to promote liver metastases in CRC [25], while chronic inflammation in the lungs from pulmonary diseases and tobacco smoking can change the lung microenvironment in a way that facilitates the establishment of pre-metastatic niches [26]. Differences in prevalence of mutations and molecular features are also observed in patients with CRC with metastases to different distant sites, with 55% of patients with CRC liver metastasis having a RAS mutation [27]. Also, patients with *BRAF* mutations are less likely to have metastases to the lung and liver than metastases to other distant sites (i.e. peritoneum) [28]. Heterogeneity in molecular features and mutational status of CRC by racial and ethnic group have been previously observed [13, 14], and may partly contribute to differences in sites and patterns of *de novo* metastases.

Prognosis of metastatic CRC varies considerably by site and pattern of metastasis. Patients with metastases isolated to a single distant site have better survival than patients with synchronous distant metastases, and metastases to the liver or lung are associated with better survival than metastases to the bone or brain [29, 30]. Furthermore, early detection of metastases has important implications for treatment and survival. In the context of liver metastases, liver resection has the potential to be curative; however, the majority of patients with CRC with liver metastases will have unresectable metastases [31]. In our cohort, surgery at the primary tumor site also differs by site of *de novo* metastasis, with patients presenting with liver metastases at diagnosis being more likely to have surgery at the primary site than patients with other sites of *de novo* metastases, and by age, with surgery more common among younger patients. Thus, identifying groups with a predisposition for specific metastatic sites can inform surveillance to identify metastatic spread early.

To our knowledge, this is the first analysis to examine disparities in site and pattern of *de novo* metastatic CRC by both age and six racial and ethnic groups. The main strength of the present study is its use of a large population-based sample spanning 10 years, which provided strong statistical power to examine associations within strata of race, ethnicity, and age. However, despite the large sample size, we are still underpowered to examine heterogeneity within broad racial and ethnic categories, such as Hispanic and API groups, where there may be differences by specific ethnic group or nativity. Additionally, our use of registry data precludes adjustment for lifestyle (such as smoking status, diet, comorbidities), individual-level socioeconomic, and screening variables which could confound the relationship between racial and ethnic group and risk of presenting with metastatic CRC. While we adjusted for county-level median household income and urban/rural county residence, we were unable to more finely adjust for socioeconomic status, which may have resulted in some residual confounding. Finally, as the SEER program does not collect data on recurrence or disease progression, we were unable to assess how risk of developing metastases after diagnosis with stage I–III CRC may differ by race, ethnicity, and age, so our analysis was limited to *de novo* metastases.

Overall, we identified groups with a greater likelihood of experiencing specific sites and patterns of *de novo* metastatic CRC. These findings can provide evidence for the prioritization of specific population groups for earlier CRC screening and for surveillance in patients with non-advanced CRC.

## Data Availability

The data used for this article is publicly accessible with completion of a data use agreement at seer.cancer.gov/data/access.html. Data analysis scripts will be shared on reasonable request to the corresponding author.
